# Assessing the long-term viability of farmers' collectives in South India

**DOI:** 10.3389/fsoc.2024.1415725

**Published:** 2024-07-29

**Authors:** Veena Suresh, S. Vivek, S. S. Sreejith

**Affiliations:** ^1^Department of Management Studies, National Institute of Technology, Calicut, Kozhikode, India; ^2^Department of Social Work, Amrita Vishwa Vidyapeetham, Coimbatore, India

**Keywords:** Farmer's Producer's Organizations, organizational sustainability, agribusiness organization, farmers collectives, rural farmers, sustainable agriculture

## Introduction

Farmers' Producer Organizations (FPOs) are collectives or associations of farmers who come together voluntarily to improve agricultural production (Hellin et al., 2009; Basavaraj et al., 2022). FPOs have a scope to transform agribusiness (Kumar Joshi and Choudhary, 2018; Prasad and Prateek, 2019; Kadam, 2022) and achieve sustainable development (Ma et al., 2023). However, there have been studies that reported the challenges faced by existing FPOs (Yadav et al., 2018; Trivedi et al., 2023), which might be an indication of the unforeseen sustainability challenges that can be expected in the journey of promotion of FPOs shortly. Hence, a critical evaluation at this point would help the key stakeholders of FPOs to systematically increase the number of FPOs in the country. The paper assesses the strengths and areas of improvements of FPOs to attain long-term viability. The evaluation areas enable similar small and marginal farmers' agribusiness organizations globally to assess the long-term viability of their farmer's collectives.

## Strength of farmers' collectives

FPOs help farmers reduce their burden of problems by collective action (Kumar and Reddy, 2023). FPOs act as platforms to double farmer's income (Mukherjee et al., 2018; Gautam and Mallaiah, 2024). FPO enabled economic, social and political empowerment of rural women (Mukherjee et al., 2019). As per Ramappa and Yashashwini (2018) study, the strength and success of FPOs depend on organizational fitness which is the upgradation of products, business and skill set of members on a timely basis. Some FPOs adopt indigenous strategies to ensure their collective action and organizational sustainability (Suresh and Sreejith, 2023). Moreover, the support of promoting agencies to handhold FPOs during the planning stage of formation enabled the development of strategies to advance FPO functions and guarantee improved earnings for the producers (Anand, 2022). Despite the short-term success case stories of FPOs there has been increasing concern on the long-term viability of FPOs.

FPO has to overcome challenges to raise capital to maximize the benefits to the members (Bikkina et al., 2018) and face constraints in operationalizing the managerial, economic and marketing activities (Singh M. et al., 2023). A lack of finance, infrastructure, and current technologies hamper farmers' growth and sustainability (Trivedi et al., 2023). FPO's functioning is compromised by weak organizational governance, ineffective leadership, and internal conflicts (Ramappa and Yashashwini, 2018). Some FPOs are being disadvantaged by a lack of processing and procurement systems and improper input supply (Nithya and Vaishnavi, 2022; Chaudhary, 2023; Radadiya and Lad, 2024). Some studies report the struggles faced by existing FPOs to resolve various organizational challenges (Bikkina et al., 2018; Yadav et al., 2018) which might be an indication of the unforeseen challenges that can be expected in the journey of promotion of FPOs shortly. Therefore, this paper aims to contextualize the challenges faced by FPOs in Kerala, India in detail as follows:

## Areas of improvement for farmers' collectives

**#1 Lack of consistent managerial competency-building programs for FPO members**

FPOs experience difficulties in achieving organizational expectations due to the poor managerial competency of members (Padmaja et al., 2019). FPO members lack the entrepreneurial skills to run the FPOs according to government-prescribed guidelines (Trebbin and Hassler, 2012). It is noticed that FPOs lack a proper business plan to upscale their business activities (Syamkrishnan, 2022). There were challenges related to the members' lack of professional expertise in organizational governance, lack of direction in building business plans, and documentation skills (Amitha et al., 2021). Possibly the most important but less talked about difficulty is to gain the trust and capabilities of FPO members to control, run and grow FPO as an agribusiness organization to accomplish the objective of doubling farmers income (Sai Krishna et al., 2021). Moreover, FPOs' functioning is compromised by weak organizational governance, ineffective leadership, and internal conflicts (Ramappa and Yashashwini, 2018). However, effective need-based capacity-building programs play a crucial role in mitigating these managerial competencies deficits in the FPO context.

A quantitative study among eighty office bearers of twelve FPOs in North India highlights six core managerial competencies—competency for planning and business development, operations control and management competency, marketing and fiancé management and democratic leadership competency for the effective performance of FPOs (Kumari, 2023). Evidence-based findings from 24 FPOs in four states of India (Maharashtra, Andhra Pradesh, West Bengal and Gujarat) revealed that continuous capacity-building programs of stakeholders were the only option for transformation of FPOs to realize their goals (Rani et al., 2023). A study conducted by a team of NABARD officers in four states of India including Kerala states confirms the significance of consistent capacity-building programs for FPO members enable effective business management of FPOs (Chintala and Mani, 2022). There is evidence of improvement in farmers' managerial performance due to training (Dola and Noor, 2011; Sahoo et al., 2024). Sunil et al. (2021) and Bharti and Kumari (2024) state that capacity-building programs on business management practices were essential to orient rural farmers about managerial skills. Therefore, our third argument is, that consistent managerial competency-building programs for FPO members enhance organizational performance and longevity of FPOs.

**#2 Limitation of Promoting Institutions to Handhold FPOs**

The support and handholding of FPOs by the key promoting institutions like SFAC (Small Farmer Agribusiness Consortium) and NABARD (National Bank for Agriculture and Rural Development) enables the smooth functioning of FPOs (Shah and Singh, 2019; Sravan et al., 2023). These institutions help farmers envision the different possibilities of the FPO (Sowmya and Raju, 2017). A study by NABARD reveals that technical handholding assistance enhances better functioning of FPOs (NABARD, 2018). An FPO in Maharashtra assessed the effectiveness of extension strategies of promoting institutions leading to competitiveness of FPOs using Asset-Process- Performance framework reveals that deployment of extension strategies with a blend of good governance and professional management leads to business competitiveness of FPOs (Wadkar, 2022).

However, the major obstacles faced by these promoting institutions are the unpredictable interference and policy changes from the government especially in the case of cash crops and grains, where government policies significantly impact market dynamics (Kloeppinger-Todd and Sharma, 2010). Additionally, a lack of specialized agri-finance experts at local banks hinders the awareness, accessibility and timely availability of financial services provided by NABARD and SFAC to the FPO members. Furthermore, the geographical distribution of FPOs across India is uneven (Senthil Nathan and Palanichamy, 2021). The diverse and complex nature of the agribusiness landscape, with its commodity-specific and regional nuances, makes it difficult for SFAC and NABARD to develop a standardized one-fit approach. Tailoring interventions to the unique needs of different FPOs requires significant time, resources and coordination. Despite these challenges sometimes the farmers themselves are less aware and not interested in availing the services due to administrative hurdles in applying and benefiting from such services. Therefore, the researcher argues that promoting institutions need to develop innovative strategies to address the uniqueness of FPOs across India through a community-based support system for effective utilization of services offered by the promoting institutions.

**#3 Inadequate Technical infrastructure and digital literacy of farmers to access digital marketing platforms**

Several studies have pointed out the positive impact of national digital marketing platforms like electronic-National Agricultural Markets (e-NAM) to sell FPO products (Kumar, 2019; Rai, 2023; Samantaray et al., 2023). Yet, there were hindrances to the implementation of e-NAM (Meena et al., 2019). Some studies mentioned the inadequate digital infrastructure challenges faced by farmers in general to access digital platforms effectively (Chadha, 2020; Smidt and Jokonya, 2022; Abate et al., 2023). Gupta and Badal (2018) captures implementation challenges of e-NAM in terms of infrastructure, institution and information. Reddy (2018) argues that elements such as commodities, farmers, traders, market organizations and quality, e-market success and competition with other players determine the success of e-markets. Another study by Raju et al. (2022) states that a medium level of knowledge on the functioning of e-NAM was significantly influenced by education, market, income and risk orientation, extension contact, mass media exposure and social participation of farmers. Moreover, there were recommendations about considering the digital climate of a place before planning strategies to bridge digital divide in farming community (Upadhyaya et al., 2019). Hence, it is significant to understand the feasibility and scope of digital marketing platforms among Kerala FPOs as well.

A study that categorized the states of India based on the level of adoption of e-NAM in agricultural marketing claims Kerala under category III where the marketing practices are predominantly regulated by cooperative societies with less participation in e-commerce (Hemayadav, 2017). This trend is visible from the e-NAM portal website statistics about the transformation of the aspirational district's program in which the number of mandis (A trading hub or market for agricultural produce) registered from Kerala is one compared to ten mandis from the neighboring state of Tamil Nadu. A survey of 220 farmers in central Kerala indicates difficulties in storage facilities, lack of computer literacy, fear of farmers adopting new technology out of ignorance and perishable nature of products as major challenges for adopting e-commerce in agriculture (Shibi and Aithal, 2022).

Thus, the first argument researchers propose is the inadequate technical infrastructure and digital literacy of farmers act as hindrances to the effective utilization of digital marketing platforms like e-NAM profitably and sustainably by farmers collectives.

## Conclusion

Assessing the long-term viability of farmer's collectives is a dynamic and continuous process. However, the three key areas can be taken into consideration by key stakeholders in the agriculture sector and similar farmer's collectives in developing countries to systematically improve the performance of farmer's collectives and achieve sustainable agriculture production.

Firstly, the lack of consistent managerial competency-building programs for FPO members needs to be addressed to enhance the organizational performance of FPOs. Despite their knowledge of agriculture, farmers often do not possess the business skills required to run a cooperative. Collectives must create fully developed, continuous training programs in operating business, financial management, marketing and organizational leadership to ensure the sustainable growth of FPOs.

Secondly the limitations of Promoting Institutions to handhold FPOs continuously need to be addressed through a community-based support system. While Promoting Institutions are essential to the initial creation and growth of an FPO, over time due to funding limitations or changing policy needs they rarely have ongoing active roles in FPO management. This makes many FPOs highly susceptible, especially during the growing phase. To address this gap and to provide continuous guidance for FPOs, a more sustainable community support system—through public-private partnerships or dedicated government initiatives, should be put in place.

Lastly, digital marketing platforms and other technological advancements in agriculture are a long way from being accessible to all farmers because of the poor technical infrastructure that prevails in many rural areas, as well as limited digital literacy among farmers. The lack of connectivity makes FPOs less competitive in an ever more digital world. It is significant to invest in digital infrastructure in rural areas and appoint tailored programs for farmers—enabling farmer collectives with wider market access deliveries, and improved operational efficiencies.

Further, responses to these key areas of improvement will rely on a partnership approach between government bodies; non-governmental organizations; and the private sector. Improving managerial competencies, continued institutional support and closing the digital divide are areas where a significant gain can be made in improving the sustainability of farmers' collectives over the long term.

## Author contributions

VS: Writing – original draft, Writing – review & editing. SV: Writing – original draft. SS: Writing – review & editing.
